# Parental Knowledge, Attitudes and Practices About Streptococcal Pharyngotonsillitis and Antibiotic Use in Western Greece

**DOI:** 10.3390/antibiotics15020149

**Published:** 2026-02-02

**Authors:** Evangelia Schortsaniti, Georgia Kourlaba, Athanasios Michos, Vana Spoulou, Gabriel Dimitriou, Despoina Gkentzi

**Affiliations:** 1 Department of Paediatrics, Patras Medical School, University General Hospital of Patras, 265 04 Patras, Greece; eviisxor@med.uoa.gr (E.S.); gdim@upatras.gr (G.D.); 2 Master of Sciences (MSc) Program “General Pediatrics and Pediatric Subspecialties: Clinical Practice and Recearch”, Medical School, National and Kapodistrian University of Athens, 115 27 Athens, Greece; 3 Department of Nursing, National and Kapodistrian University of Athens, 115 27 Athens, Greece; kurlaba@nurs.uoa.gr; 4 Division of Infectious Diseases, First Department of Pediatrics, Medical School, National and Kapodistrian University of Athens, “Aghia Sophia” Children’s Hospital, 115 27 Athens, Greece; amichos@med.uoa.gr; 5 Immunobiology and Vaccinology Research Laboratory, Department of Infectious Diseases, “Aghia Sophia” Children’s Hospital, 115 27 Athens, Greece; vspoulou@med.uoa.gr; 6 First Department of Pediatrics, Medical School, National and Kapodistrian University of Athens, 115 27 Athens, Greece

**Keywords:** Group A Streptococcus, streptococcal infection, pharyngitis, parental knowledge, attitudes, practices, invasive infection

## Abstract

**Background/Objectives**: *Streptococcus pyogenes* (Group A beta-hemolytic streptococcus, GAS) is the most common cause of bacterial pharyngitis and a major contributor to morbidity and mortality worldwide. There has been an increase in invasive GAS infections and related deaths in several European countries post-COVID-19 pandemic. We aimed to assess parental knowledge, attitudes, and practices regarding GAS pharyngotonsillitis, with a focus on antibiotic use and misuse. **Methods**: A cross-sectional questionnaire-based study was conducted on a convenience sample of parents of children admitted to the Pediatric Ward or visiting the Pediatric Emergency Department of the University General Hospital of Patras, Greece (September 2024–February 2025). For knowledge assessment, the questionnaire consisted of 10 True/False questions, based on which a total knowledge score was calculated. For attitude and practice assessment, the questionnaire consisted of 10 Likert scale questions. A Negative Practice Score was calculated as a sum of the answers in five practices with a negative perspective, with higher scores indicating worse practices and lower scores indicating better practices. **Results**: The study enrolled 378 parents, 79% of them were aware that not all children with a sore throat need antibiotics, and 61% believed asymptomatic children with a positive strep antigen test should receive antibiotics. Concerns about GAS transmissibility were high (76%), while attitudes about severity were mixed. A median GAS total knowledge score of 6 (IQR: 4–7) indicated moderate knowledge. Multivariable analysis revealed that male parents, non-immigrants, those previously hospitalized for GAS infection, and those informed by pediatricians or reliable websites had significantly higher knowledge scores. Regarding practices, most parents (72%) disagreed with requesting antibiotics from pediatricians, and 93.9% did not administer leftover antibiotics. Additionally, 58% expressed more concerns in recent years due to the increase in invasive infections. The median GAS Negative Practice Score was 10.5 (IQR: 7.0–13.0), indicating generally good practices, as lower scores correspond to fewer negative practices. Older parents and those with higher knowledge scores were also linked to fewer negative practices in multivariable analysis. **Conclusions**: These findings highlight the importance of targeted education on GAS pharyngotonsillitis and the need to focus on specific population groups to reduce antibiotic misuse.

## 1. Introduction

Pharyngotonsillitis is one of the most common reasons for seeking medical advice in pediatrics [1]. Even though most of these episodes are of viral origin, approximately 5–30%, depending on age, are due to GAS infection [1,2]. GAS infection can result in serious complications and is responsible for half a million deaths worldwide each year, mainly due to rheumatic heart disease and invasive disease [1,3]. In recent years, post-COVID-19 pandemic, there has been an increase in the number of cases of invasive GAS infection in many European countries, especially in children under 10 years of age [4,5,6,7,8]. Consequently, parents and clinicians are more alert, and they are changing their daily practices with regard to diagnosis and antibiotic use [9].

In the absence of a vaccine that can prevent infection, timely diagnosis and appropriate use of antibiotics are the cornerstones of appropriate management [3]. The current rapid diagnostic tests are qualitative, not quantitative, and they indicate only the presence or absence of GAS. As a result, they cannot differentiate carriage from infection, which may lead to unnecessary antibiotic prescriptions in patients whose symptoms are due to viral infection but are GAS carriers [10]. The shortage of amoxicillin-based antibiotics, starting in October 2022, due to the increased prescription of such antibiotics, is notable [9,11]. This is probably due to parents worrying about the prompt diagnosis of GAS infection to prevent its evolution into an invasive one. However, there is no evidence yet to support that invasive infection is caused by untreated GAS pharyngotonsillitis [6,9]. Published data so far suggest that this surge is likely due to a variety of other reasons, including a parallel increase in respiratory viruses or infection from the varicella zoster virus [4,6,9,12].

In the case of GAS pharyngotonsillitis, antibiotics help to prevent complications, speed up remission of symptoms, and reduce transmission [13,14,15]. Between 5 and 15% of school-age children are asymptomatic carriers, and there is no evidence to suggest that carriage puts individuals or others at higher risk if left untreated [15]. However, carriage causes a significant diagnostic challenge and often results in unnecessary antibiotic use, which leads to adverse effects, places a financial burden on the healthcare system and contributes to antibiotic resistance [16,17]. Antibiotic resistance is a global issue and among the top 10 threats to public health according to the World Health Organization [18]. Greece is one of the countries in Europe with the highest rates of antibiotic use in the community and of resistant bacterial infections in the European Union [16,19]. One of the reasons doctors prescribe antibiotics unnecessarily is the belief that patients expect antibiotics for their illness. However, the quality of communication between parents and doctors plays an important role, as parents seem to remain satisfied, despite not being given antibiotics, if the physician explains to them the reason for not prescribing them [20,21].

Several studies have examined parental views on antibiotic use for upper respiratory tract infections. One such study conducted in our country by Panagakou et al. in 2011 showed that most parents had a trusting relationship with their pediatrician and would not choose another physician based on whether antibiotics were prescribed [22]. Although 80% of parents knew that most upper respiratory tract infections are self-limiting, 74% still expected antibiotics to be prescribed. Only 10% administered antibiotics without medical advice, and 80% knew that unnecessary antibiotic use leads to antibiotic resistance [21].

A review of the literature found only one study that focused on GAS pharyngotonsillitis. This study was conducted in Utah, United States, in 1987 on 120 parents visiting the Pediatric Department of a University Hospital and a private clinic in Salt Lake City [23]. In that study, parents scored highest on treatment-related questions and lowest on those concerning diagnosis. Notably, 87% of parents believed that most tonsillitis episodes are bacterial in origin, only 25% were aware of possible complications of strep throat, and 30% did not know that GAS is transmitted between family members. The main source of knowledge for parents was their previous contact with the disease, health professionals, and friends and relatives, while only 16% were informed by the mass media of the time [23]. However, this study had several limitations, including a small sample number of participants, collected from two departments homogeneous in terms of ethnicity and income (Anglo-American, middle class). Furthermore, it was conducted at a time when the use of social media was limited, and internet use was not as widespread, both of which are, at present, important sources of information for parents [23,24].

Therefore, in the current era, there is a need for accurate recording of knowledge, attitudes, and misconceptions regarding the prevention and management of GAS pharyngotonsillitis and subsequent antimicrobial use. GAS pharyngotonsillitis warrants focused attention because it is the only bacterial upper respiratory infection for which a rapid diagnostic test exists, allowing antibiotics to be prescribed appropriately based on test results and medical examination. Special focus should be given to identifying knowledge gaps among parents in order to guide educational interventions where they are most needed. In this study, we aim to evaluate the knowledge, attitudes and practices of parents regarding GAS pharyngotonsillitis. Secondarily, we aim to evaluate if parent age, gender, educational level, and socio-economic status and the media they use affect their knowledge and attitudes and if the increase in invasive streptococcal infections altered their attitudes.

## 2. Results

### 2.1. Primary Objectives

Out of 491 questionnaires distributed, 380 were returned (response rate, 77.3%). Two questionnaires were excluded later because of poor completion. Table 1 demonstrates the sociodemographic characteristics of the population surveyed in our study.

The median (Q1–Q3) total knowledge score was 6 (4–7), indicating moderate knowledge from the study participants.

Among the 378 participating parents, 56.2% incorrectly believed that pharyngotonsillitis is mainly caused by bacteria, and 29.9% identified GAS as the most common cause. Regarding the rapid strep test, 70.6% believed it should be performed on all children with pharyngotonsillitis, while 66.7% considered a positive test in a child with pharyngotonsillitis to be confirmation of GAS etiology. Furthermore, 66.6% of parents misidentified viral symptoms as streptococcal features. Although 79% recognized that not all children with pharyngotonsillitis should get antibiotics, 61.4% believed that all children with a positive strep test, even if asymptomatic, should receive antibiotic treatment. In total, 68.8% identified throat culture as the gold standard for GAS pharyngotonsillitis, while 61.1% knew that GAS pharyngotonsillitis is more common in school-aged children.

Descriptives for knowledge sources are presented in Table 2.

**Figure 1:**
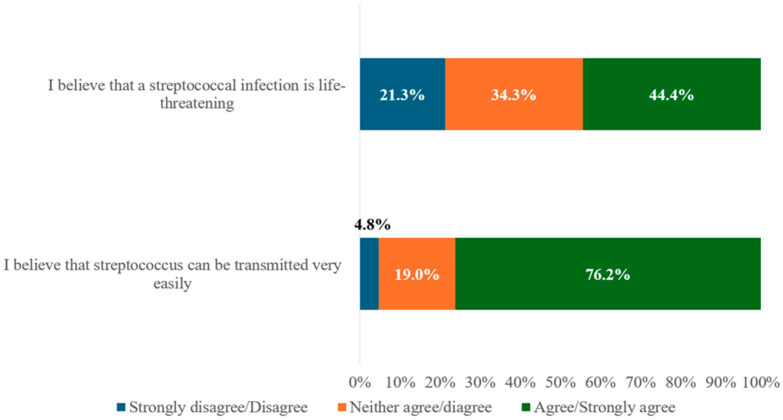
Descriptives for parental attitudes about GAS.

Descriptives for answers regarding attitudes are presented in Figure 1.

**Figure 2:**
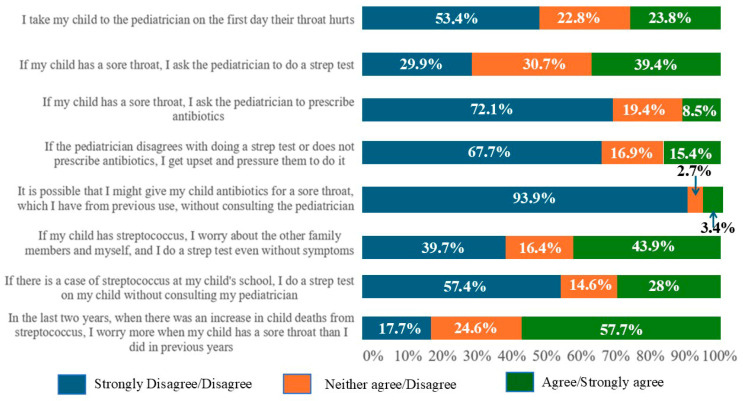
Descriptives for parental practices relevant to GAS.

Descriptives for the answers in the practice questionnaire are presented in Figure 2. The median (Q1–Q3) Negative Practice Score was 10.5 (7.0–13.0), indicating a below-average level of agreement regarding negative practices among the study participants.

### 2.2. Secondary Objectives

#### 2.2.1. Total Knowledge Score and Factors Affecting It

Table 3 shows the factors affecting the total knowledge score.

#### 2.2.2. Negative Practices and Factors Affecting It

Table 4 shows the factors affecting the negative practices. In the multivariable analysis, the participant’s age and the total knowledge score appeared to be associated with the Negative Practice Score.

Regarding factors associated with agreement with the practice “*If my child has a sore throat*, *I ask the pediatrician to prescribe antibiotics*”, the results are presented in Supplemental Table S1. In the multivariable analysis, only the total knowledge score appeared to be associated with the level of agreement with this practice.

As for agreement with the practice “*If the pediatrician disagrees with doing a strep antigen test or does not prescribe antibiotics*, *I get upset and pressure them to do it*”, the results are presented in Supplemental Table S2.

Finally, regarding the statement “*It is possible that I might give my child antibiotics for a sore throat*, *which I have from previous use*, *without consulting the pediatrician*”, sex, family income, and being informed through non-reliable websites or social media appeared to be associated with the level of agreement with this practice in the multivariate analysis.

## 3. Discussion

This study provides new insights into parental knowledge, attitudes, and practices regarding Group A Streptococcal (GAS) pharyngotonsillitis in a large sample of Greek parents. Although this is, to our knowledge, the first study to examine this topic in a Greek clinical sample, the findings should not be generalized to the wider population. Our results suggest a generally adequate level of knowledge within this hospital-based sample, with private pediatricians serving as the primary and most trusted source of information. Nonetheless, misconceptions about the causes and management of pharyngitis persist, highlighting the ongoing need for targeted educational interventions.

In this study, half of the parents achieved a knowledge score above six, indicating moderate knowledge within this specific sample, rather than reflecting all Greek parents. However, detailed analysis revealed several misconceptions. Nearly half believed that most throat infections are bacterial, despite correctly identifying that GAS is not the most common cause. This may reflect confusion between bacteria and viruses due to a lack of information in the field. Most parents were also unable to correctly identify viral sore throat symptoms. A majority believed that a strep antigen test should be performed on all children with a sore throat and that a positive result indicates GAS as the cause of symptoms. Although most understood that not all GAS-positive children require antibiotics, many worryingly believed that GAS carriage should be treated—possibly due to unfamiliarity with the concept of asymptomatic carriage. This is in clear contrast with established guidelines, which state that antimicrobial therapy is not indicated for the vast majority of GAS carriers and should be considered only in a small number of specific situations, such as outbreaks of acute rheumatic fever or invasive GAS, outbreaks in closed communities, a personal or family history of acute rheumatic fever, marked parental anxiety, or when tonsillectomy is being considered solely due to carriage [25]. Furthermore, more than half cited their private pediatrician as their primary source of information, indicating a potential association between parental reliance on healthcare professionals and trust in their guidance. Nonetheless, others relied on personal experience (22.0%), both reliable (23.9%) and non-reliable online sources (22.8%), and friends or relatives (28.0%). This underscores the need for accessible, evidence-based public health communication in the field. In terms of attitudes, almost half of the parents viewed GAS infection as life-threatening, and the vast majority believed that it was highly contagious. This likely reflects the increased concern in recent years after the COVID-19 pandemic, following the publicity around this area. Finally, regarding practices, most parents showed appropriate behavior around antibiotic use, neither requesting nor pressuring for prescriptions, and few endorsed using antibiotics without medical advice.

The present study also identified several factors that influence parental knowledge and practices regarding GAS pharyngotonsillitis. Multivariable analysis indicated that sex, immigrant status, hospitalization for GAS pharyngotonsillitis, working in healthcare, vaccination site, age of children, and the quality of information sources were associated with the total knowledge score, even though a broader range of variables appeared significant in the univariate analysis, such as age, education, income, area of residence, and the age of the firstborn child. Many of these lost their significance after adjusting for confounders. This suggests that some associations were likely mediated by more direct influences, such as access to reliable healthcare information or personal health experiences. Importantly, being informed by a personal/private pediatrician, working in healthcare, having been hospitalized for GAS infection, or using trustworthy online sources seemed to have a positive effect on the total knowledge score. These findings suggest a potential link between the use of credible sources and knowledge. These findings also reflect the structure of the primary healthcare system in Greece, where pediatric care is predominantly provided by private practice pediatricians rather than general practitioners. Children are regularly followed by pediatricians who are well trained in common childhood illnesses, including GAS pharyngitis, which may be associated with parental reliance on pediatricians for information. This model differs from that of some other European countries, where general practitioners often serve as the first point of contact for pediatric care. The strong role of pediatricians in Greece likely contributes to the high percentage of parents relying on them for knowledge. Furthermore, while both the public and private fields provide pediatric care, the private field remains the main setting for routine child health monitoring.

Regarding negative practices, multivariable analysis showed that older age and higher knowledge scores were associated with more appropriate practices (i.e., lower Negative Practice Scores), indicating a potential relationship between experience, knowledge, and fewer anxiety-driven behaviors. While several sociodemographic factors (e.g., education, income, parental status, and ethnicity) appeared significant in univariate models, most lost significance in the multivariable analysis. This suggests that targeted educational interventions may be associated with improved parental practices regardless of background. Importantly, knowledge was the only factor that remained significantly associated with the likelihood of requesting antibiotics, highlighting its key role in supporting rational antibiotic use. In contrast, no variable significantly predicted dissatisfaction or pressure when antibiotics were not prescribed. Single parents were more likely to administer leftover antibiotics without consultation, while males and those with higher knowledge scores were less likely to do so. The use of unreliable information sources (e.g., social media) was also associated with poorer practices, underlining the need for clear, evidence-based health communication—particularly for groups who may rely on such sources due to limited trust in the healthcare system or other barriers to reliable information.

Most of these results are in agreement with the previous study conducted in Utah on GAS pharyngotonsillitis, as most parents answered correctly regarding questions about the transmission of GAS, diagnosis through throat culture swab, and the fact that it is more common among school-aged children [23]. Parents in this study were also confused about the bacterial or viral origin of the majority of sore throats, and almost half of them did not correctly recognize that a runny nose and cough are symptoms of viral tonsillitis. Regarding demographic characteristics, our sample had similar distributions in terms of sex, age, marital status, income, education, number of children, and ethnicity. In that previous study, prior experience, social networks, and healthcare professionals were reported as the primary sources of knowledge regarding GAS pharyngotonsillitis, all with similarly high proportions. In the present study, parents most frequently cited their private pediatrician as their main source of information, indicating sustained trust in healthcare professionals. Unlike earlier findings, digital sources now represent a significant component of parental knowledge, as expected. This shift may potentially reflect improved access to healthcare services and the increasing influence of digital media in health-related decision-making and indicates the importance of critically assessing and developing reliable online health resources. It is also important to guide parents towards evidence-based sources of information, as some still rely on social media platforms, which may not always provide accurate or reliable health content. Additionally, in the 1987 study, the only factor found to affect knowledge among those examined was marital status. Our study identified additional factors that may influence parental knowledge. Furthermore, attitudes and practices were not examined in that study.

Regarding antibiotics, our findings are consistent with those of the study conducted by Panagakou et al. in 2011 in Greece, as most parents do not administer antibiotics without medical advice, and only a small percentage agree that they would request an antibiotic prescription for an upper respiratory tract infection [22]. General knowledge and misconceptions about GAS pharyngotonsillitis may have remained unchanged over the years, possibly suggesting that public education efforts on upper respiratory infections have not significantly improved or reached all population groups. Furthermore, parents probably still rely primarily on their personal/private pediatrician’s advice, despite the recent increase in invasive GAS infections and the accompanying rise in parental anxiety.

The findings of this study highlight several suggestions for future practice and research in the field of parental management of GAS pharyngotonsillitis. Given that parental knowledge was associated with better practices, which may be linked to lower antibiotic misuse, educational interventions should focus on addressing common misconceptions held by parents. For instance, many parents believe that most sore throats are bacterial or that symptoms of viral infections are caused by GAS. Additionally, such programs should provide a clear explanation of GAS carriage. As being informed by a pediatrician and using reliable online sources were both positively associated with higher knowledge and better practices, healthcare systems should empower pediatricians or other primary healthcare providers to act as primary educators for parents. Developing evidence-based, easy-to-understand materials for pediatricians to share with families, and promoting the use of validated online resources, could further improve outcomes. Special attention should be given to vulnerable groups, such as Roma, immigrants, and single parents, as univariate analysis showed less knowledge and worse practices among these populations. Interestingly, the total knowledge score (in the multivariable analysis) and practices were not influenced by the vaccination site (public vs. private healthcare) as we had expected, likely suggesting that pediatricians, regardless of the setting, make an equal effort to inform parents. A significant number of parents still expect a strep antigen test whenever their child has a sore throat or perform one when in doubt. Therefore, detailed clinical indications for testing should be communicated by pediatricians to help reduce overdiagnosis and inappropriate antibiotic use. The generally low agreement with giving antibiotics without medical consultation is encouraging. Of note, in Greece, by law in 1973 and then in 2020 with a stricter law, antibiotics are not allowed to be dispensed from pharmacies without a medical prescription, as it used to happen in the past [26]. However, continued efforts are needed to reinforce antibiotic stewardship, particularly by addressing misconceptions, such as the need to treat GAS carriage. Campaigns should clearly define when antibiotics are appropriate and emphasize the potential risks of overuse.

These findings have direct implications for antimicrobial stewardship in Greece. These GAS-specific misconceptions highlight practical targets for stewardship, guiding interventions on when testing is needed and how antibiotic prescriptions should be communicated. Furthermore, the strong reliance on private pediatricians positions them as the most effective channel for intervention, particularly in counseling parents on when testing is appropriate and when antibiotics are truly indicated. The strict prescription-only rules for antibiotics in Greece help prevent inappropriate access to antibiotics, but our findings show that parental misconceptions—especially about testing and GAS carriage—can still affect how parents seek care and what they expect during a medical visit. Feasible stewardship strategies arising from our results include developing brief, evidence-based materials for pediatricians, improving access to validated online resources, and developing communication strategies that effectively reach vulnerable groups (e.g., immigrants, single parents, and families relying on social media). Strengthening parental understanding in these areas could potentially further enhance the effectiveness of existing antibiotic regulations and support national efforts to reduce unnecessary use.

This study is not without limitations. First, a convenience sample was used, consisting of parents of children who visited the Pediatric Emergency Department at the University Hospital of Patras or were hospitalized on the pediatric ward. This may introduce sample bias, as parents who visit hospitals may differ significantly from those who do not, both in terms of knowledge and other sociodemographic factors. Therefore, the results should be generalized with caution. Furthermore, the internal consistency of the total knowledge score and Negative Practice Score was moderate (KR-20 and Cronbach’s alpha values ranging from 0.65 to 0.68), which should be taken into account when interpreting these scores. In addition, the questionnaire was not explicitly guided by a formal behavioral theory; the selection of items and the construction of total knowledge scores and Negative Practice Scores were informed primarily by our clinical observations and by review of existing questionnaires, rather than by an established theoretical framework, which may limit the generalizability of the findings to other contexts or populations. Nevertheless, this approach ensures that the questionnaire reflects actual parental behavior regarding GAS pharyngitis and provides actionable insights for antibiotic stewardship in practice. Furthermore, data were collected through self-completed questionnaires, which may have led to misconceptions or biases from the parents. It is also possible that the total knowledge score or practices were overestimated, as parents who agreed to participate in the study may have been more confident about the topic. Many parents who declined to participate or did not return the questionnaire mentioned that they lacked information on this subject. The study was conducted approximately two years after the outbreak of invasive GAS infections, meaning that for some questions, parents were asked to recall their knowledge or practices from some time ago. In addition, some knowledge items—particularly those related to asymptomatic carriage or test interpretation—may have been challenging for parents less familiar with medical terminology. This represents a limitation, as some responses may have limited exposure to these concepts. Although the study was conducted at the University Hospital of Patras, which treats patients from the broader Achaia region, it remains geographically limited. Parents from less accessible areas may differ from those in urban settings. Lastly, while parents’ knowledge was assessed through specific True/False questions, their practices were evaluated using Likert scale questions based on their perceptions of their behavior. These responses may not fully reflect actual behavior, as they could be influenced by social expectations or the desire to appear to demonstrate “better” or “appropriate” behavior.

## 4. Materials and Methods

A cross-sectional prospective study was conducted from September 2024 to February 2025 on a convenience sample of parents (either fathers or mothers) of children who were admitted to the General Pediatric Ward of University General Hospital of Patras or who visited the Pediatric Emergency Department of the same hospital. The parents chosen were adults, inhabitants of the regional unit of Achaia (one of the largest prefectures in Greece with an estimated number of 306.000 inhabitants), and had at least one child of 16 years of age or less. They were handed, face to face, a questionnaire, an overview of the study objectives and a written consent form.

The questionnaire was developed following a literature search and included 34 questions divided into two parts [1,3,9,10,13,20,21,23,27]. The first part collected sociodemographic information, and the second part assessed parental knowledge, attitudes and practices regarding GAS pharyngotonsillitis. To ensure clarity, content validity and internal consistency, it was piloted with 25 parents and adjusted accordingly.

The sample size was estimated taking into consideration the number of adult parents living in Achaia who have at least one child 16 years old or less and assuming that 50% of participants will have a satisfactory level of knowledge (at least a score of 5) with a margin of error of 5% and a confidence interval of 95%.

The study was approved by the Research Ethics Committee of the University General Hospital of Patras and the National and Kapodistrian University of Athens (7 August 2024 and 26 September 2024 respectively).

For the assessment of knowledge, the questionnaire consisted of 10 True/False questions (1: correct answer, 0: incorrect answer/“I don’t know”), based on which a total knowledge score was calculated (sum of all the correct answers with possible values from 0 to 10). For the assessment of attitudes, the questionnaire consisted of 2 Likert scale questions (1: strongly disagree; 2: disagree; 3: neither agree/disagree; 4: agree; 5: strongly agree). For the assessment of practices, the questionnaire consisted of 8 Likert scale questions (1: strongly disagree; 2: disagree; 3: neither agree/disagree; 4: agree; 5: strongly agree). A Negative Practice Score was calculated as a sum of the answers in five practices with a negative perspective: “If my child has a sore throat, I ask the pediatrician to prescribe antibiotics”; “If the pediatrician disagrees with doing a strep antigen test or does not prescribe antibiotics, I get upset and pressure them to do it”; “If my child has streptococcus, I worry about the other family members and myself, and I do a strep antigen test even without symptoms”; “If there is a case of streptococcus at my child’s school, I do a strep antigen test on my child without consulting my pediatrician”; “It is possible that I might give my child antibiotics for a sore throat, which I have from previous use, without consulting the pediatrician” (possible scores from 5 to 25, with higher scores indicating worse practices). The remaining three practices (“I take my child to the pediatrician on the first day their throat hurts”; “If my child has a sore throat, I ask the pediatrician to do a strep antigen test”; “In the last two years, when there was an increase in child deaths from streptococcus, I worry more when my child has a sore throat than I did in previous years”) were recoded as binary outcome variables (0: strongly disagree/disagree/neither agree/disagree; 1: agree/strongly agree) and assessed separately.

Continuous variables were summarized with mean along with standard deviation (SD), median along with 1st–3rd quartiles (Q1–Q3), and min–max. Categorical variables were summarized with absolute (n) and relative (%) frequencies. The internal reliability of the knowledge questionnaire was assessed using the Kuder–Richardson Formula 20 (KR-20), while the internal reliability of the negative practices was assessed using Cronbach’s alpha. Both the knowledge and the negative practices questionnaires had moderate–borderline internal reliability, with a KR-20 value of 0.65 and a Cronbach’s alpha of 0.68, respectively.

Linear regression models were applied to identify socio-economic factors and information sources associated with the parents’ knowledge, while robust linear regression* was applied to identify socio-economic factors and information sources associated with the negative practices regarding tonsillopharyngitis. Logistic regression models were applied for the assessment of the three remaining practices. Univariate models were initially fitted, and factors with a *p*-value less than 0.15 were included in the multivariable models. Possible covariates tested were socio-economic factors (participant’s age, sex, number of children, age of firstborn child, education level, whether the participant was an immigrant or a Roma, area of residence, monthly family income, children’s vaccinations’ usual site, single parent, healthcare professional, previous hospitalization for streptococcal infection) and knowledge sources (personal/private pediatrician, experience with GAS pharyngotonsillitis, working in healthcare, reliable websites, other websites, mass media, friends/relatives, educational institutions, general knowledge). For the Negative Practice Score and the three remaining practices, the total knowledge score was also tested as a possible significant factor.

The significance level for all analyses was set at 5%. All data management and analyses were conducted using the statistical program STATA v.17.0.

## 5. Conclusions

This study revealed that, while parents generally have a moderate level of knowledge regarding GAS pharyngotonsillitis, several misconceptions persist, particularly concerning the cause and diagnosis of the infection. Specifically, many parents believe that all sore throats should be tested for GAS and that a positive result always indicates the need for antibiotic treatment. On the other hand, the majority of parents appeared to understand that antibiotics should not be administered indiscriminately, although there is some uncertainty regarding the concept of GAS carriage. In addition, some parents performed strep antigen tests even though it was unnecessary, and half of them reported increased worry over the past few years, possibly associated with the increase in invasive infections. This study identified several factors that were associated with parental knowledge and practices—such as age, immigration status, being hospitalized for GAS infection, and those informed by a pediatrician—and age and total knowledge score respectively. While most parents demonstrated positive practices regarding antibiotic use, concerns remain about unnecessary tests and the overuse of antibiotics, particularly in vulnerable groups. This study underscores the need for further educational programs, especially targeting vulnerable population groups, and emphasizes the importance of reliable information from healthcare professionals, such as pediatricians. To better understand the underlying causes of parental misconceptions and inappropriate practices, future research should involve larger, more representative samples and focus on the development of educational programs that may enhance parental understanding of respiratory infections and appropriate antibiotic use. However, as this study was based on a hospital-based convenience sample, the findings may not be fully generalizable to all parents in Greece.

## Figures and Tables

**Table 1:** Descriptives for demographics.

Patient Characteristics, n (%)	N = 378
Sex	N = 378
Male	80 (21.2%)
Female	298 (78.8%)
Participant’s age, years	N = 377
18–29	42 (11.1%)
30–44	257 (68.2%)
>45	78 (20.7%)
Number of children	N = 378
1	117 (31.0%)
2	183 (48.4%)
3	56 (14.8%)
4 or more	22 (5.8%)
Age of firstborn child, years	N = 375
<2 years	52 (13.9%)
≥2 years	323 (86.1%)
Immigrant	N = 376
Yes	21 (5.6%)
No	355 (94.4%)
Roma	N = 377
Yes	18 (4.8%)
No	359 (95.2%)
Area of residence	N = 374
Rural	42 (11.2%)
Semi-urban	72 (19.3%)
Urban	260 (69.5%)
Education	N = 371
Elementary/Middle school	43 (11.6%)
High school	140 (37.7%)
University	188 (50.7%)
Family income, monthly	N = 299
<500 €	23 (7.7%)
500–1000 €	67 (22.4%)
1000–3000 €	159 (53.2%)
>3000 €	50 (16.7%)
Children’s vaccinations’ usual site	N = 372
Private healthcare	321 (86.3%)
Public healthcare	51 (13.7%)
Single parent	N = 376
Yes	30 (8.0%)
No	346 (92.0%)
Healthcare professional	N = 376
Yes	31 (8.2%)
No	345 (91.8%)
Hospitalized for streptococcal infection	N = 378
Yes	5 (1.3%)
No	373 (98.7%)

**Table 2:** Descriptives for GAS knowledge sources.

Knowledge Sources, n (%)	N = 372
Personal/private pediatrician	189 (50.8%)
Previous experience with streptococcal pharyngotonsillitis	82 (22.0%)
Working in healthcare	27 (7.3%)
Reliable websites on the internet, e.g., Department of Hellenic Public Health (DoHPH), World Health Organization (WHO)	89 (23.9%)
Other websites on the internet/social media	85 (22.8%)
Mass media (television, radio)	63 (16.9%)
Friends/relatives	104 (28.0%)
Educational institutions	2 (0.5%)
General knowledge	7 (1.9%)

Each participant may have reported more than one knowledge source.

**Table 3:** Factors associated with the total knowledge score.

	Univariate Models		Multivariable Model	
	Coef. (95% CI)	*p*	Coef. (95% CI)	*p*
Participant’s Age	N = 377		N = 281	
30–44 vs. 18–29	1.66 (0.93, 2.39)	<0.001	0.06 (−0.95, 1.08)	0.862
>45 vs. 18–29	1.56 (0.72, 2.40)		−0.11 (−1.24, 1.03)	
Sex	N = 378		N = 281	
Female vs. male	0.87 (0.31, 1.43)	0.002	0.78 (0.22, 1.35)	0.007
Number of children	N = 378		N = 281	
2 vs. 1	0.71 (0.18, 1.24)	0.069	0.47 (−0.18, 1.11)	0.434
3 vs. 1	0.41 (−0.32, 1.13)		0.48 (−0.33, 1.30)	
4 or more vs. 1	0.60 (−0.44, 1.63)		0.77 (−0.36, 1.90)	
Age of firstborn child	N = 375		N = 281	
≥2 years vs. <2 years	1.14 (0.48, 1.80)	0.001	0.43 (−0.39, 1.26)	0.301
Education level	N = 371		N = 281	
High school vs. elementary/middle	1.17 (0.41, 1.94)	<0.001	0.17 (−1.10, 1.44)	0.435
University vs. elementary/middle	1.82 (1.08, 2.56)		0.50 (−0.80, 1.80)	
Immigrant	N = 376		N = 281	
Yes vs. no	−1.88 (−2.87, −0.88)	<0.001	−1.46 (−2.80, −0.12)	0.032
Roma	N = 377		N = 281	
Yes vs. no	−2.28 (−3.34, −1.22)	<0.001	−1.78 (−3.60, 0.04)	0.055
Area of residence	N = 374		N = 281	
Semi-urban vs. rural	−0.55 (−1.41, 0.31)	0.018	0.07 (−0.88, 1.02)	0.586
Urban vs. rural	0.30 (−0.44, 1.04)		0.33 (−0.49, 1.14)	
Family income, monthly	N = 299		N = 281	
500–1000 € vs. <500 €	−0.17 (−1.20, 0.86)	<0.001	−1.18 (−2.34, −0.03)	0.077
1000–3000 € vs. <500 €	1.00 (0.05, 1.95)		−0.57 (−1.73, 0.59)	
>3000 € vs. <500 €	1.42 (0.35, 2.49)		−0.36 (−1.63, 0.90)	
Children’s vaccinations’ usual site	N = 372		N = 281	
Public healthcare vs. private healthcare	−0.80 (−1.47, −0.13)	0.019	−0.26 (−1.07, 0.55)	0.528
Single parent	N = 376		N = 281	
Yes vs. no	−0.79 (−1.64, 0.06)	0.070	0.23 (−0.72, 1.17)	0.640
Healthcare professional	N = 376		N = 281	
yes vs. no	1.57 (0.75, 2.40)	<0.001	−0.56 (−2.36, 1.25)	0.545
Hospitalized for streptococcal infection	N = 378		N = 281	
yes vs. no	3.05 (1.05, 5.05)	0.003	3.23 (0.40, 6.06)	0.025
Knowledge sources (each vs. no)	N = 372		N = 281	
Personal/private pediatrician	1.00 (0.54, 1.45)	<0.001	0.63 (0.13, 1.13)	0.013
Experience with GAS pharyngotonsillitis	0.74 (0.19, 1.30)	0.009	0.29 (−0.31, 0.90)	0.340
Working in healthcare	1.88 (1.00, 2.75)	<0.001	2.35 (0.52, 4.18)	0.012
Reliable websites (DoHPH, WHO)	1.25 (0.72, 1.78)	<0.001	0.68 (0.11, 1.25)	0.019
Other websites (internet/social media)	−0.22 (−0.78, 0.33)	0.426		
Mass media (television, radio)	−0.38 (−1.00, 0.24)	0.232		
Friends/relatives	−0.02 (−0.54, 0.50)	0.940		
Educational institutions	1.10 (−2.06, 4.25)	0.497		
General knowledge	−0.56 (−2.27, 1.15)	0.519		

Linear regression was performed with the dependent variable, the total knowledge score. Variables with a significance level of *p* < 0.15 in the univariable analyses were considered for entry into the multivariable model. Statistical significance was determined using *p*-values derived from Wald chi-square tests, with a two-sided significance level. CI: Confidence Interval.

**Table 4:** Factors associated with Negative Practice Score.

	Univariate Models		Multivariable Model	
	Coef. (95% CI)	*p*	Coef. (95% CI)	*p*
Participant’s Age	N = 375		N = 284	
30–44 vs. 18–29	−3.84 (−5.15, −2.54)	<0.001	−2.34 (−4.32, −0.37)	0.049
>45 vs. 18–29	−4.15 (−5.66, −2.65)		−2.66 (−4.84, −0.48)	
Sex	N = 376		N = 284	
Female vs. male	−0.69 (−1.73, 0.35)	0.192		
Number of children	N = 376		N = 284	
2 vs. 1	−1.65 (−2.60, −0.70)	0.005	−0.78 (−2.02, 0.46)	0.256
3 vs. 1	−1.56 (−2.86, −0.26)		−1.23 (−2.81, 0.35)	
4 or more vs. 1	−0.55 (−2.41, 1.31)		0.45 (−1.70, 2.59)	
Age of firstborn child	N = 373		N = 284	
≥2 years vs. <2 years	−1.69 (−2.91, −0.48)	0.006	−0.51 (−2.09, 1.07)	0.525
Education level	N = 369		N = 284	
High school vs. elementary/middle	−1.70 (−3.11, −0.29)	<0.001	1.59 (−0.86, 4.05)	0.290
University vs. elementary/middle	−2.64 (−4.01, −1.28)		1.02 (−1.49, 3.53)	
Immigrant	N = 374		N = 284	
Yes vs. no	1.99 (0.15, 3.82)	0.034	0.69 (−1.92, 3.30)	0.604
Roma	N = 377		N = 284	
Yes vs. no	3.49 (1.52, 5.46)	0.001	2.71 (−0.85, 6.27)	0.135
Area of residence	N = 372		N = 284	
Semi-urban vs. rural	0.20 (−1.37, 1.77)	0.070	0.44 (−1.38, 2.26)	0.598
Urban vs. rural	−0.94 (−2.28, 0.41)		−0.18 (−1.74, 1.38)	
Family income, monthly	N = 298		N = 284	
500–1000 € vs. <500 €	−0.22 (−2.17, 1.73)	0.005	1.41 (−0.83, 3.66)	0.295
1000–3000 € vs. <500 €	−2.12 (−3.92, −0.32)		0.37 (−1.83, 2.57)	
>3000 € vs. <500 €	−1.70 (−3.73, 0.33)		0.99 (−1.41, 3.40)	
Children’s vaccinations’ usual site	N = 370		N = 284	
Public healthcare vs. private healthcare	0.68 (−0.56, 1.92)	0.279		
Single parent	N = 374		N = 284	
Yes vs. no	1.64 (0.09, 3.18)	0.038	0.33 (−1.46, 2.12)	0.715
Healthcare professional	N = 374		N = 284	
Yes vs. no	−3.47 (−4.97, −1.97)	<0.001	−1.75 (−5.26, 1.76)	0.328
Hospitalized for streptococcal infection	N = 376		N = 284	
Yes vs. No	−1.11 (−4.88, 2.66)	0.563		
Knowledge sources (each vs. no)	N = 371		N = 284	
Personal/private pediatrician	−0.93 (−1.77, −0.08)	0.032	−0.27 (−1.25, 0.71)	0.584
Experience with GAS pharyngotonsillitis	−0.74 (−1.78, 0.29)	0.160		
Working in healthcare	−3.87 (−5.51, −2.24)	<0.001	−1.80 (−5.40, 1.81)	0.328
Reliable websites (DoHPH, WHO)	−0.63 (−1.63, 0.37)	0.218		
Other websites (internet, social media)	0.95 (−0.06, 1.96)	0.066	0.31 (−0.80, 1.41)	0.583
Mass media (television, radio)	0.85 (−0.29, 1.99)	0.143	0.04 (−1.19, 1.26)	0.952
Friends/relatives	−0.08 (−1.03, 0.87)	0.867		
Educational institutions	4.01 (−1.81, 9.84)	0.176		
General knowledge	1.35 (−1.79, 4.49)	0.398		
Total knowledge score	N = 376		N = 284	
	−0.54 (−0.72, −0.37)	<0.001	−0.28 (−0.51, −0.054)	0.016

Robust linear regression with the dependent variable, the Negative Practice Score. Variables with a significance level of *p* < 0.15 in the univariable analyses were considered for entry into the multivariable model. Statistical significance was determined using *p*-values derived from Wald chi-square tests, with a two-sided significance level. CI: Confidence Interval. For the Negative Practice Score, lower values indicate better practices, and higher values indicate worse practices.

## Data Availability

The dataset is available upon request from the authors.

## Supplementary Materials

The following supporting information can be downloaded at: https://www.mdpi.com/article/10.3390/antibiotics15020149/s1, Table S1: Factors associated with agreement to the practice “If my child has a sore throat, I ask the pediatrician to pre-scribe antibiotics”. Table S2: Factors associated with agreement to the practice “If the pediatrician disagrees with doing a strep antigen test or does not prescribe antibiotics, I get upset and pressure them to do it”.

## Abbreviations

The following abbreviations are used in this manuscript:

GAS	Group A Streptococcus
SD	Standard Deviation
KR-20	Kuder–Richardson Formula 20
CI	Confidence Interval
DoHPH	Department of Hellenic Public Health
WHO	World Health Organization

## References

[1] Pellegrino R., Timitilli E. (2023). Acute Pharyngitis in Children and Adults: Descriptive Comparison of Current Recommendations from National and International Guidelines and Future Perspectives. Eur. J. Pediatr..

[2] Miller K.M., Carapetis J.R. (2022). The Global Burden of Sore Throat and Group A *Streptococcus pharyngitis*: A Systematic Review and Meta-Analysis. EClinicalMedicine.

[3] Brouwer S., Rivera-Hernandez T. (2023). Pathogenesis, Epidemiology and Control of Group A Streptococcus Infection. Nat. Rev. Microbiol..

[4] Guy R., Henderson K.L. (2023). Increase in Invasive Group A Streptococcal Infection Notifications, England, 2022. Eurosurveillance.

[5] CDC Increase in Invasive Group A Strep Infections, 2022–2023. https://archive.cdc.gov/www_cdc_gov/groupastrep/igas-infections-investigation.html.

[6] Increase in Invasive Group A Streptococcal Infections Among Children in Europe, Including Fatalities. https://www.who.int/europe/news/item/12-12-2022-increase-in-invasive-group-a-streptococcal-infections-among-children-in-europe--including-fatalities.

[7] De Gier B., Marchal N. (2023). Increase in Invasive Group A Streptococcal (*Streptococcus pyogenes*) Infections (IGAS) in Young Children in the Netherlands, 2022. Eurosurveillance.

[8] Lassoued Y., Assad Z. (2023). Unexpected Increase in Invasive Group A Streptococcal Infections in Children After Respiratory Viruses Outbreak in France: A 15-Year Time-Series Analysis. Open Forum Infect. Dis..

[9] Mariani F., Gentili C. (2023). State of the Art of Invasive Group A Streptococcus Infection in Children: A Scoping Review of the Literature with a Focus on Predictors of Invasive Infection. Children.

[10] Sabharwal K.A., Simon M.W. (2023). Are Clinicians Overdiagnosing Strep Throat and Overprescribing Antibiotics?. Front. Pediatr..

[11] European Medicines Agency Shortage of Amoxicillin and Amoxicillin/Clavulanic Acid Various Presentations Including Paediatric Formulations and Presentations. http://www.ema.europa.eu/contact.

[12] Thacharodi A., Hassan S. (2024). The Burden of Group A Streptococcus (GAS) Infections: The Challenge Continues in the Twenty-First Century. iScience.

[13] Fox J.W., Marcon M.J. (2006). Diagnosis of Streptococcal Pharyngitis by Detection of *Streptococcus pyogenes* in Posterior Pharyngeal versus Oral Cavity Specimens. J. Clin. Microbiol..

[14] Urkin J., Allenbogen M. (2013). Acute Pharyngitis: Low Adherence to Guidelines Highlights Need for Greater Flexibility in Managing Paediatric Cases. Acta Paediatr. Int. J. Paediatr..

[15] Speert D.P. (1998). Group A Streptococcal Carriage: Can the Troll Be Tamed?. Paediatr. Child Health.

[16] Gourgoulis G.M., Katerelos P. (2013). Antibiotic Prescription and Knowledge about Antibiotic Costs of Physicians in Primary Health Care Centers in Greece. Am. J. Infect. Control.

[17] Oikonomou M.-E., Gkentzi D. (2021). Parental Knowledge, Attitude, and Practices on Antibiotic Use for Childhood Upper Respiratory Tract Infections during COVID-19 Pandemic in Greece. Antibiotics.

[18] Antimicrobial Resistance. https://www.who.int/news-room/fact-sheets/detail/antimicrobial-resistance.

[19] Barmpouni M., Gordon J.P. (2023). Clinical and Economic Value of Reducing Antimicrobial Resistance in the Management of Hospital-Acquired Infections with Limited Treatment Options in Greece. Infect. Dis. Ther..

[20] Ong S., Nakase J. (2007). Antibiotic Use for Emergency Department Patients With Upper Respiratory Infections: Prescribing Practices, Patient Expectations, and Patient Satisfaction. Ann. Emerg. Med..

[21] Vinker S., Ron A. (2003). The Knowledge and Expectations of Parents about the Role of Antibiotic Treatment in Upper Respiratory Tract Infection-a Survey among Parents Attending the Primary Physician with Their Sick Child Upper Respiratory Tract Infection—A Survey Among Parents Attending the Primary Physician with Their Sick Child. BMC Fam. Pract..

[22] Panagakou S.G., Papaevangelou V. (2011). Antibiotic Use for Upper Respiratory Tract Infections in Children: A Cross-Sectional Survey of Knowledge, Attitudes, and Practices (KAP) of Parents in Greece. BMC Pediatr..

[23] Hillard S.K. (1987). Streptococcal Pharyngitis: What Do Parents Know?. Master’s Thesis.

[24] Frey E., Bonfiglioli C. (2022). Parents’ Use of Social Media as a Health Information Source for Their Children: A Scoping Review. Acad. Pediatr..

[25] Shulman S.T., Bisno A.L., Clegg H.W., Gerber M.A., Kaplan E.L., Lee G., Martin J.M., Van Beneden C. (2012). Clinical Practice Guideline for the Diagnosis and Management of Group A Streptococcal Pharyngitis: 2012 Update by the Infectious Diseases Society of America. Clin. Infect. Dis..

[26] Kopsidas I., Kokkinidou L. (2023). Dispensing of Antibiotics without Prescription in the Metropolitan Area of Athens, Greece, in 2021—Can New Legislation Change Old Habits?. Antimicrob. Steward. Healthc. Epidemiol. ASHE.

[27] Panagakou S.G., Theodoridou M.N. (2009). Development and Assessment of a Questionnaire for a Descriptive Cross—Sectional Study Concerning Parents’ Knowledge, Attitudes and Practises in Antibiotic Use in Greece. BMC Infect. Dis..

