# Supplementary material for: Parental Knowledge, Attitudes and Practices About Streptococcal Pharyngotonsillitis and Antibiotic Use in Western Greece
Source: Antibiotics (Basel). 2026 Feb 2;15(2):149. doi: 10.3390/antibiotics15020149 (PMC12937277; doi:10.3390/antibiotics15020149)
Supplement: Supplementary file 1 [file antibiotics-15-00149-s001.zip › antibiotics-4005916-supplementary.pdf]

antibiotics

MDPI

Supplemental Table S1 : Factors associated with agreement to the practice “If my child has a sore throat, I ask the pediatrician to prescribe antibiotics”

	Univariate models		Multivariable model	
	OR (95% CI)	p	OR (95% CI)	p
Participant’s age	N=376		N=289	
30 – 44 vs. 18 – 29	0.23 (0.10, 0.54)	**0.002**	0.91 (0.20, 4.08)	0.563
> 45 vs. 18 – 29	0.22 (0.07, 0.70)		0.39 (0.05, 2.98)	
Sex	N=377		N=289	
Female vs. Male	0.65 (0.29, 1.47)	0.301		
Number of children	N=377		N=289	
2 vs. 1	1.31 (0.54, 3.17)	0.851		
3 vs. 1	1.64 (0.54, 4.96)			
4 or more vs. 1	1.36 (0.27, 6.89)			
Age of firstborn child	N=374		N=289	
≥ 2 years vs. < 2 years	2.47 (0.57, 10.70)	0.225		
Education level	N=370		N=289	
High school vs. Elementary/Middle	0.23 (0.09, 0.61)	**0.001**	0.42 (0.07, 2.61)	0.394
University vs. Elementary/Middle	0.19 (0.07, 0.48)		0.26 (0.04, 1.86)	
Immigrant	N=375		N=289	
Yes vs. No	2.74 (0.86, 8.70)	0.087	1.05 (0.13, 8.26)	0.966
Roma	N=376		N=289	
Yes vs. No	8.48 (3.02, 23.77)	**< 0.001**	3.90 (0.42, 36.27)	0.231
Area of residence	N=373		N=289	
Semi-urban vs. Rural	1.19 (0.33, 4.21)	0.488		
Urban vs. Rural	0.71 (0.23, 2.21)			
Family income, monthly	N=298		N=289	
500 – 1000 € vs. < 500 €	0.34 (0.10, 1.13)	**0.013**	1.31 (0.22, 7.84)	0.897
1000 – 3000 € vs. < 500 €	0.15 (0.05, 0.48)		0.99 (0.15, 6.54)	
> 3000 € vs. < 500 €	0.18 (0.04, 0.80)		1.66 (0.19, 14.18)	
Children vaccinations' usual site	N=371		N=289	
Public healthcare vs. Private	1.88 (0.77, 4.60)	0.168		
Single parent	N=375		N=289	
Yes vs. No	2.45 (0.87, 6.94)	0.091	0.61 (0.11, 3.29)	0.561
Healthcare professional	N=375		N=289	
Yes vs. No	0.34 (0.04, 2.55)	0.292		
Hospitalized for streptococcal infection	N=377		N=289	
Yes vs. No	2.75 (0.30, 25.37)	0.372		
Knowledge sources (Each vs. No)	N=372		N=289	
Personal/private pediatrician	0.84 (0.41, 1.74)	0.642		
Experience with GAS pharyngotonsillitis	0.34 (0.10, 1.15)	0.083	0.56 (0.11, 2.81)	0.482

**Working in healthcare**	0.39 (0.05, 2.97)	0.363		
**Reliable websites (DoHPH, WHO)**	0.71 (0.28, 1.80)	0.475		
**Other websites (internet, Social media)**	1.14 (0.49, 2.64)	0.762		
**Mass media (Television, Radio)**	1.42 (0.59, 3.44)	0.438		
**Friends / Relatives**	1.01 (0.45, 2.26)	0.982		
**Educational institutions**				
**General Knowledge**	1.80 (0.21, 15.40)	0.593		
**Total Knowledge Score**	**N=377**		**N=289**	
	0.69 (0.59, 0.81)	**< 0.001**	0.79 (0.63, 0.99)	**0.041**

Logistic regression was performed with dependent variable the agreement the practice "If my child has a sore throat, I ask the pediatrician to prescribe antibiotics" (0: Strongly disagree/Disagree/Neither agree/disagree; 1: Agree/Strongly agree). Variables with a significance level of $p < 0.15$ in the univariable analyses were considered for entry into the multivariable model. Statistical significance was determined using p-values derived from Wald chi-square tests, with a two-sided significance level.CI: Confidence Interval

Supplemental Table S2: Factors associated with agreement to the practice "If the pediatrician disagrees with doing a strep antigen test or does not prescribe antibiotics, I get upset and pressure them to do it"

	Univariate models		Multivariable model	
	OR (95% CI)	p	OR (95% CI)	p
**Participant's age**	**N=377**		**N=293**	
**30 – 44 vs. 18 – 29**	0.38 (0.18, 0.79)	**0.007**	0.67 (0.22, 2.03)	0.461
**> 45 vs. 18 – 29**	0.22 (0.08, 0.61)		0.41 (0.10, 1.70)	
**Sex**	**N=378**		**N=293**	
**Female vs. Male**	1.81 (0.82, 4.00)	0.140	1.41 (0.55, 3.58)	0.476
**Number of children**	**N=378**		**N=293**	
**2 vs. 1**	0.47 (0.24, 0.92)	**0.030**	0.59 (0.25, 1.43)	0.125
**3 vs. 1**	1.18 (0.54, 2.59)		1.70 (0.65, 4.45)	
**4 or more vs. 1**	1.62 (0.57, 4.61)		1.72 (0.44, 6.66)	
**Age of firstborn child**	**N=375**		**N=293**	
**≥ 2 years vs. < 2 years**	0.85 (0.39, 1.86)	0.693		
**Education level**	**N=371**		**N=293**	
**High school vs. Elementary/Middle school**	0.58 (0.25, 1.36)	0.226		
**University vs. Elementary/Middle school**	0.48 (0.21, 1.10)			
**Immigrant**	**N=376**		**N=293**	
**Yes vs. No**	1.78 (0.63, 5.07)	0.280		
**Roma**	**N=377**		**N=293**	
**Yes vs. No**	4.94 (1.86, 13.13)	**0.001**	2.02 (0.42, 9.70)	0.380
**Area of residence**	**N=374**		**N=293**	
**Semi-urban vs. Rural**	1.20 (0.41, 3.48)	0.926		
**Urban vs. Rural**	1.06 (0.42, 2.68)			
**Family income, monthly**	**N=299**		**N=293**	
**500 – 1000 € vs. < 500 €**	0.55 (0.19, 1.61)	**0.041**	0.85 (0.24, 3.08)	0.700
**1000 – 3000 € vs. < 500 €**	0.27 (0.10, 0.76)		0.56 (0.16, 1.98)	
**> 3000 € vs. < 500 €**	0.25 (0.07, 0.91)		0.57 (0.13, 2.57)	
**Children vaccinations' usual site**	**N=372**		**N=293**	
**Public healthcare vs. Private healthcare**	1.01 (0.45, 2.27)	0.984		
**Single parent**	**N=376**		**N=293**	
**Yes vs. No**	1.45 (0.56, 3.71)	0.443		
**Healthcare professional**	**N=376**		**N=293**	
**Yes vs. No**	0.17 (0.02, 1.26)	0.083	0.40 (0.05, 3.19)	0.384
**Hospitalized for streptococcal infection**	**N=378**		**N=293**	
**Yes vs. No**	3.77 (0.62, 23.10)	0.151		
**Knowledge sources (Each vs. No)**	**N=372**		**N=293**	
**Personal/private pediatrician**	0.89 (0.51, 1.55)	0.675		

**Experience with GAS pharyngotonsillitis**	0.80 (0.39, 1.63)	0.539		
**Working in healthcare**				
**Reliable websites (DoHPH, WHO)**	0.71 (0.35, 1.43)	0.337		
**Other websites (internet, Social media)**	1.83 (0.99, 3.38)	0.053	1.52 (0.71, 3.25)	0.284
**Mass media (Television, Radio)**	1.35 (0.67, 2.72)	0.408		
**Friends / Relatives**	1.08 (0.58, 2.01)	0.803		
**Educational institutions**	5.46 (0.34, 88.49)	0.233		
**General Knowledge**	0.90 (0.11, 7.62)	0.923		
**Total Knowledge Score**	**N=378**		**N=293**	
	0.88 (0.78, 1.00)	**0.044**	0.92 (0.78, 1.09)	0.353

Logistic regression was performed with dependent variable the agreement the practice “If the pediatrician disagrees with doing a strep antigen test or does not prescribe antibiotics, I get upset and pressure them to do it” (0: Strongly disagree/Disagree/Neither agree/disagree; 1: Agree/Strongly agree). Variables with a significance level of $p < 0.15$ in the univariable analyses were considered for entry into the multivariable model. Statistical significance was determined using p-values derived from Wald chi-square tests, with a two-sided significance level.CI: Confidence Interval
